# Optimization of Collagenase Production by *Pseudoalteromonas* sp. SJN2 and Application of Collagenases in the Preparation of Antioxidative Hydrolysates

**DOI:** 10.3390/md15120377

**Published:** 2017-12-02

**Authors:** Xinghao Yang, Xiao Xiao, Dan Liu, Ribang Wu, Cuiling Wu, Jiang Zhang, Jiafeng Huang, Binqiang Liao, Hailun He

**Affiliations:** 1 School of Life Sciences, Central South University, Changsha 410013, China; 132511003@csu.edu.cn (X.Y.); 152501023@csu.edu.cn (X.X.); 162501022@csu.edu.cn (D.L.); 152511020@csu.edu.cn (R.W.); 152511020@csu.edu.cn (C.W.); jiangzhang915@csu.edu.cn (J.Z.); 1608110217@csu.edu.cn (J.H.); 162511005@csu.edu.cn (B.L.); 2 Hunan Bailin Biological Technology Incorporated Company, Changsha 410205, China

**Keywords:** collagenase, fermentation optimization, collagen, *Pseudoalteromonas*, antioxidant peptides

## Abstract

Collagenases are the most important group of commercially-produced enzymes. However, even though biological resources are abundant in the sea, very few of these commercially popular enzymes are from marine sources, especially from marine bacteria. We optimized the production of marine collagenases by *Pseudoalteromonas* sp. SJN2 and investigated the antioxidant activities of the hydrolysates. Media components and culture conditions associated with marine collagenase production by *Pseudoalteromonas* sp. SJN2 were optimized by statistical methods, namely Plackett–Burman design and response surface methodology (RSM). Furthermore, the marine collagenases produced by *Pseudoalteromonas* sp. SJN2 were seen to efficiently hydrolyze marine collagens extracted from fish by-products, and remarkable antioxidant capacities of the enzymatic hydrolysates were shown by DPPH radical scavenging and oxygen radical absorbance capacity (ORAC) tests. The final optimized fermentation conditions were as follows: soybean powder, 34.23 g·L^−1^; culture time, 3.72 d; and temperature, 17.32 °C. Under the optimal fermentation conditions, the experimental collagenase yield obtained was 322.58 ± 9.61 U·mL^−1^, which was in agreement with the predicted yield of 306.68 U·mL^−1^. Collagen from Spanish mackerel bone, seabream scale and octopus flesh also showed higher DPPH radical scavenging rates and ORAC values after hydrolysis by the collagenase. This study may have implications for the development and use of marine collagenases. Moreover, seafood waste containing beneficial collagen could be used to produce antioxidant peptides by proteolysis.

## 1. Introduction

Collagen is the main component of the extracellular matrix. Collagen is the predominant constituent of skin, tendons and cartilage and is the main organic component of bones, teeth and corneas [1,2]. Collagen is not only a structural protein with high tensile strength, but also a protein that affects cell differentiation, migration and attachment. Collagen is an inexpensive and resourceful meat by-product that is used extensively as a food additive to increase the texture, water-holding capacity and stability of several food products. The main sources of collagen, such as bovine and porcine skin and bone, are derived from land-based animals. Recently, components of marine organisms, including fish skin, bone and scales, have received increasing attention as sources of collagen [3]. Additionally, with collagen being the most abundant protein in all higher organisms, collagenases have diverse biotechnological applications [4,5,6].

Collagenases, a class of proteases with high specificity for collagen, can cleave peptide bonds in collagen [7]. Collagenases are usually considered virulence factors and normally target the connective tissue in muscle cells and other organs. On the other hand, collagenases can be used to produce bioactive collagen peptides, which have been widely used in the pharmaceutical, food and cosmetic industries. To date, a number of collagenases of bacterial origin have been identified and characterized, such as MCP01 from *Pseudoalteromonas* sp. SM9913 [8], Col H from *Clostridium histolyticum* [9] and collagenases from *Vibrio alginolyticus* [10]. However, the productivity of most collagenases is not high enough for industrial application. Recently, marine bacteria have become known as important sources for the identification of novel enzymes. Compared with collagenases from land animals, marine collagenases usually have higher catalytic efficiency towards marine collagen from fish skin and bone [8]. Therefore, collagenases obtained from marine bacteria in particular have received attention owing to their diverse properties. Simultaneously, there have been attempts to increase the production of marine-derived collagenases and to optimize the fermentation conditions for the production of these enzymes [11].

Statistical methods such as factorial design, central composite design and response surface methodology (RSM) have been frequently used to optimize process parameters for the production of different kinds of enzymes [12]. Statistically-designed experiments are more effective than those designed using classical optimization strategies because they can be used to study many variables simultaneously with a low number of observations, saving time and expense. The Plackett–Burman design provides a way to rapidly screen for main variables with significant effects on a specified parameter from a large number of variables, and the information obtained can be retained for further optimization [13,14]. Response surface methodology (RSM) can identify interactions among various factors while requiring less experimental resources. Therefore, RSM, as a collection of statistical techniques that are useful for designing experiments, building models, evaluating the effects of different factors and searching for optimal conditions of studied factors for predictable responses, has been successfully applied in many areas of biotechnology, including the protein enzyme industry [15]. *Pseudoalteromonas* sp. SJN2, isolated from the inshore environment of the South China Sea, can produce extracellular collagenases that have high catalytic efficiency. The aim of this study was to use RSM to optimize the fermentation medium to increase collagenase production by *Pseudoalteromonas* sp. SJN2. In addition, marine collagen extracted from seafood by-products was digested by crude extracellular collagenases secreted by *Pseudoalteromonas* sp. SJN2, and the antioxidant activity of hydrolysates was measured.

## 2. Results

### 2.1. Purification and Enzymatic Properties of Ps sp. SJN2 Collagenases

Collagenases SJN2 was sequentially purified using ammonium sulfate precipitation, anion exchange and size exclusion chromatography; Col SJN2 purification is shown in Figure S1 and Table S1 (Supplementary Material). Currently, as a highly effective feed additive, enzymes used in the food industry are crude enzymes because the cost of pure enzymes is high and because the operation is tedious [16]. In addition, combinations of enzymes can result in a range of possible biological properties for the corresponding hydrolysates.

**Figure 1:**
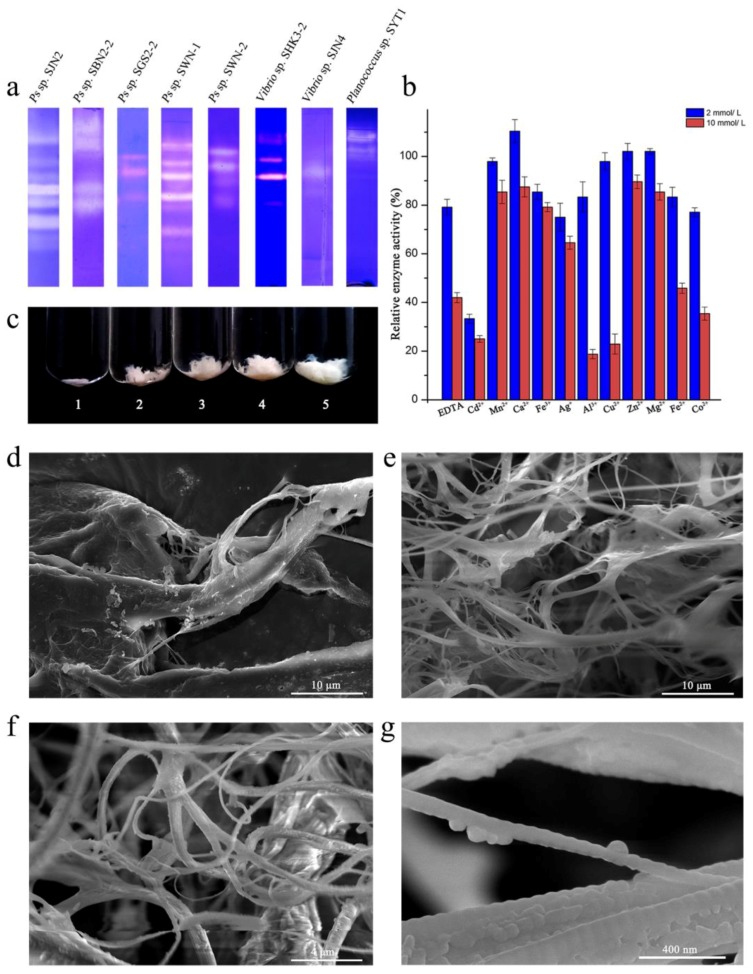
Enzymatic properties of *Pseudoalteromonas* sp. SJN2 collagenases. (**a**) Comparison of catalytic ability on gelatin of collagenases from *Ps* sp. SJN2, *Ps* sp. SBN2-2, *Ps* sp. SGS2-2, *Ps* sp. SWN-1, *Ps* sp. SWN-2, *Vibrio* sp. HK3-2, *Vibrio* sp. SJN4, and *Planococcus* sp. SYT1; (**b**) effect of ions against *Ps* sp. SJN2 collagenases’ enzyme activity, red column for 2 mM, blue column for 10 mM; (**c**) swelling effect of *Ps* sp. SJN2 collagenase on bovine collagen type I: Tube 1: 200 μL 20 mM PBS treated for 24 h at 37 °C; Tubes 2–5: 200 μL *Ps* sp. SJN2 crude enzyme treated for 1 h, 5 h, 12 h and 24 h; (**d**–**g**) swelling of insoluble collagen was observed under SEM, the (d) control group with 8000× magnification and (**e**–**g**) the experimental group with 8000×, 20,000× and 250,000× magnification.

Compared with other strains isolated from the inshore environment of the South China Sea at the same time, *Pseudoalteromonas* sp. SJN2 collagenases, which appear as bright strips in Figure 1a Line 1, have some advantages in terms of the number and brightness of the strips, which suggest a strong ability to hydrolyze collagen. 

The effects of ions on the enzymatic activities of *Ps* sp. SJN2 collagenases was measured, as shown in Figure 1b. Some ions (Zn^2+^, Mg^2+^ and Ca^2+^; at lower concentrations; red column) were seen to promote *Ps* sp. SJN2 collagenase activity or were less toxic to strain *Ps* sp. SJN2 than EDTA and Cd^2+^.

Swelling of insoluble collagen after hydrolysis by *Ps* sp. SJN2 collagenases was observed (Figure 1c–g). With increasing enzyme hydrolysis time, the porosity of the insoluble collagen increased. As observed by SEM, the collagen structure in the control group remained compact and tough, while in the experimental group, the bulky collagen fiber bundles changed into small dispersed collagen fibers (Figure 1e), and the sub-fibers (Figure 1f) were exposed, which indicated the collagen-hydrolysis ability of *Ps* sp. SJN2 collagenases.

### 2.2. Catalytic Efficiency of Collagenases from Ps sp. SJN2

**Figure 2:**
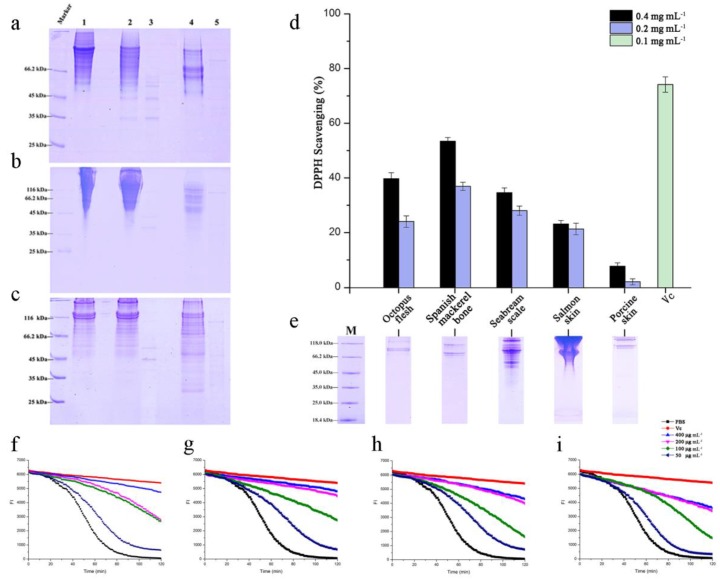
Differences between Col H and collagenases from *Ps* sp. SJN2 in the degradation of fish collagen and the antioxidative test. (**a**–**c**) Fish-collagen substrates from skin, scale and bone after hydrolysis are shown in Line 1: fish collagen; Line 2: fish collagen hydrolysed by Col H with a reaction time of 1 min; Line 3: Col H; Line 4: fish collagen hydrolysed by collagenases from *Ps* sp. SJN2 at a reaction time of 1 min; Line 5: collagenases from *Ps* sp. SJN2. (**d**,**e**) DPPH scavenging of five collagen peptides (d) and the extracted collagen (e). The reaction concentration of extracted collagen for the black column is 0.4 mg·mL^−1^, and that for the blue column is 0.2 mg·mL^−1^. The green is vitamin C at 0.1 mg·mL^−1^, as a positive control. (**f**–**i**) ORAC assay for four kinds of marine collagen peptides: (f) octopus flesh, (g) seabream scale, (h) Spanish mackerel bone and (i) salmon skin. The red curve is vitamin C at 100 μg·mL^−1^, as a positive control, while the black is PBS, as a negative control. For each substrate, the blue curve is 400 μg·mL^−1^ of hydrolysed collagen; the purple curve is 200 μg·mL^−1^; the green curve is 100 μg·mL^−1^; and the navy curve is 50 μg·mL^−1^.

Compared with commercially available terrestrial Col H, collagenases from *Ps* sp. SJN2, as enzymes from marine sources, have a competitive edge in the degradation of collagen from marine-biological sources. Figure 2a shows the hydrolysis of fish skin collagen by collagenases from *Ps* sp. SJN2 into smaller molecular collagen-peptides than those produced using Col H, after a 1-min reaction at 45 °C with both collagenases having low concentrations (2 mg·mL^−1^ Col H and 0.2 mg·mL^−1^ crude enzymes from *Ps* sp. SJN2), which indicated that crude collagenases from *Ps* sp. SJN2 had higher catalytic efficiency than Col H. In the degradation of marine collagen, marine collagenases exhibit properties such as low enzyme concentrations and rapid catalysis, and the same results can be obtained for the degradation of collagen from fish scales (Figure 2b) and fish bone (Figure 2c). This result showed that crude collagenases from *Ps* sp. SJN2 could be more suitable for the digestion of marine collagen, which was seen to be spliced into small polypeptide fragments. Improving the production of collagenases from *Ps* sp. SJN2 would be useful for future application of this enzyme.

### 2.3. Initial Screening of Significant Fermentation Conditions

The effects of temperature, initial pH, seed inoculation, culture time, corn meal concentration, bran liquid concentration and soybean meal concentration, which are the seven variables associated with collagenase production in the Plackett–Burman design, are shown in Table 1 and Table 2. A random experimental program was devised by using Design-Expert-8.0.6 software. Analysis of variance (ANOVA) results are listed in Table 3.

Mathematical analysis of this model shows that the model was significant (*F*-value = 9.77, *p*-value = 0.0219), with a complex correlation *R*^2^ = 0.9447, indicating that the model can explain 94.47% of the experimental results. The multiple regression Equation (1) describes the mathematical relationship between collagenase production and fermentation conditions:

*Y* = 198.38 − 26.16*X_1_* + 0.86*X_2_* − 11.01*X_3_* − 18.89*X_4_* + 1.45*X_5_* + 8.16*X_6_* + 17.52*X_7_* (1)

For every decrease in *X_1_*, a decrease of 26.16 units in *Y* is predicted; a similar effect is predicted for the other variables. This relationship between *Y* and *X* was further used in the steepest ascent experiment. Table 3 indicates that the terms with *p*-values less than 0.05, i.e., *X_1_* (temperature, *p* = 0.0054), *X_4_* (culture time, *p* = 0.0169) and *X_7_* (soybean meal concentration, *p* = 0.0216), had relatively significant effects on collagenase yield. Therefore, the steepest ascent experiment was designed with the three significant variables mentioned above.

The path of steepest ascent was determined based on a decrease of 2.00 °C for *X_1_* (temperature), a decrease of 0.64 d for *X_4_* (culture time) and an increase of 3.00 g·L^−1^ for *X_7_* (soybean concentration). The movement was generated along the path until no improvement of *Y* (yield of collagenases from *Ps* sp. SJN2) occurred. After the first step of the coordinates, decreasing collagenase yield was observed, as shown in Table 4. Consequently, this combination (temperature, 16 °C; culture time, 3.36 d; and soybean concentration, 33 g·L^−1^) was selected as the middle point (zero level) for a final optimal design by response surface methodology.

### 2.4. Further Optimization by Response Surface Methodology

The fermentation conditions were further explored by response surface methodology based on central composite design. The design matrix and the corresponding results of RSM are shown in Table 5 and Table 6, and the ANOVA results of the RSM are displayed in Table 7.

The adequacy of the model was assessed using ANOVA (Table 7). The coefficient of determination (*R*^2^) was 0.9694 for collagenase production, indicating good agreement between the experimental and predicted values [17]. The results demonstrated that the 96.94% variability in the response experiment could be explained by this model. The R^2^ is always between zero and 1.0, and a value closer to 1.0 indicates a stronger model and better response. A very low value of the coefficient of variation (C.V., 7.01%) indicated high reliability and precision of the experimental simulation. The *F*-value of the model was 35.19, which implied that the model was statistically significant. The smaller the *p*-value, the more significant the corresponding coefficient could be considered. The *p*-value of 0.0001, which is less than 0.05, suggested that the model terms were significant. These results indicated that temperature, culture time and soybean concentration have a direct relationship with collagenase production.

A multivariate regression model with interaction terms was characterized by the following regression Equation (2):

*Y* = 287.87 + 26.22*A* + 18.21*B* + 33.95*C* + 10.15*AB* + 1.58*AC* + 8.93*BC* − 24.74*A*^2^ − 25.18*B*^2^ − 48.46*C*^2^ (2)

where *Y* is the predicted collagenase production (U·mL^−1^) and *A*, *B* and *C* are the values of temperature (°C), culture time (d) and soybean powder concentration (g·L^−1^), respectively.

**Figure 3:**
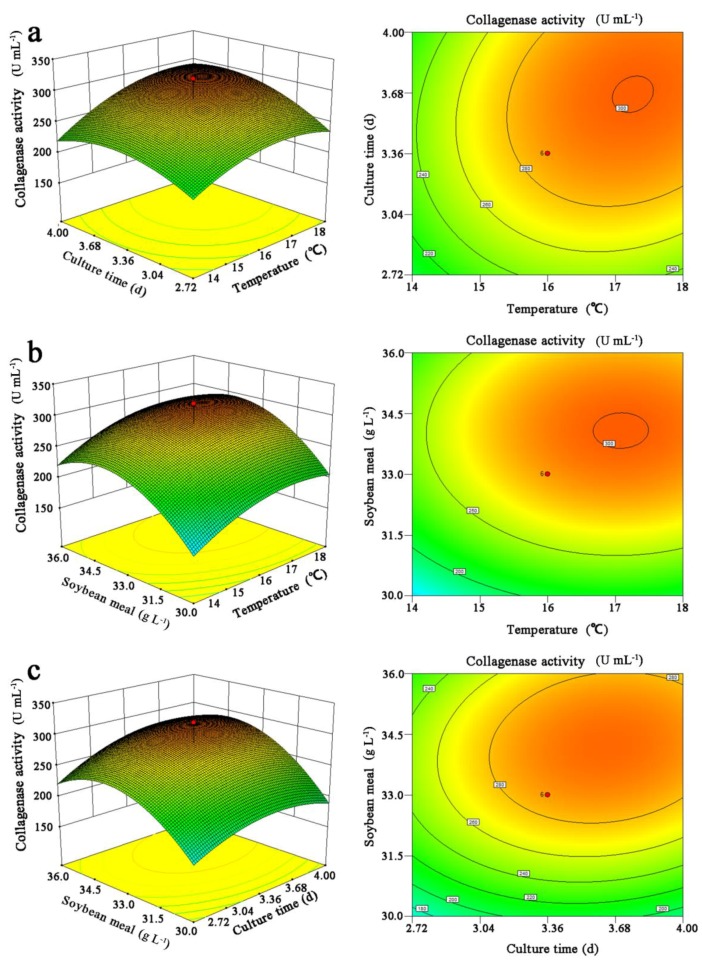
Response surface plots (left) and two-dimensional contour plots (right) of the effects of (**a**) culture time vs. temperature, (**b**) soybean concentration vs. temperature and (**c**) soybean concentration vs. culture time on the yield of collagenases from *Ps* sp. SJN2.

The three-dimensional response surface plots and two-dimensional contour plots were used to elucidate the relationship and interaction effects of the chosen fermentation conditions for maximal production of collagenases from *Ps* sp. SJN2, as shown in Figure 3. In each sketch, two variables linearly changed within the experimental range, while the other variable remained constant at the central point. For the two-dimensional contour plots, the shape of the contour described the interaction significance of the paired variables, and the elliptical appearance suggested an extremely significant interaction.

Figure 3a shows the response surface and the corresponding contour plot of culture time (*A*) vs. temperature (*B*), keeping soybean concentration (*C*) at the zero level. It can be noticed from the surface that the optimal yield of collagenases from *Ps* sp. SJN2 was observed when the culture time was approximately at the −1 level, while the temperature was nearly at the +1 level. The two-dimensional contour plot of culture time (*A*) vs. temperature (*B*) showed an elongated pattern, suggesting that the interaction between culture time and temperature has a significant effect on the yield of collagenases from *Ps* sp. SJN2. Similar profiles were also observed in Figure 3b,c.

The experimental data were fitted to Equation (2), and the optimal levels of the significant variables were determined to be as follows: culture time, 3.72 d; temperature, 17.32 °C; and soybean concentration, 34.23 g·L^−1^; with a predicted maximum production of collagenases from *Ps* sp. SJN2 at 306.683 U·mL^−1^.

### 2.5. Experimental Validation of the Model

**Figure 4:**
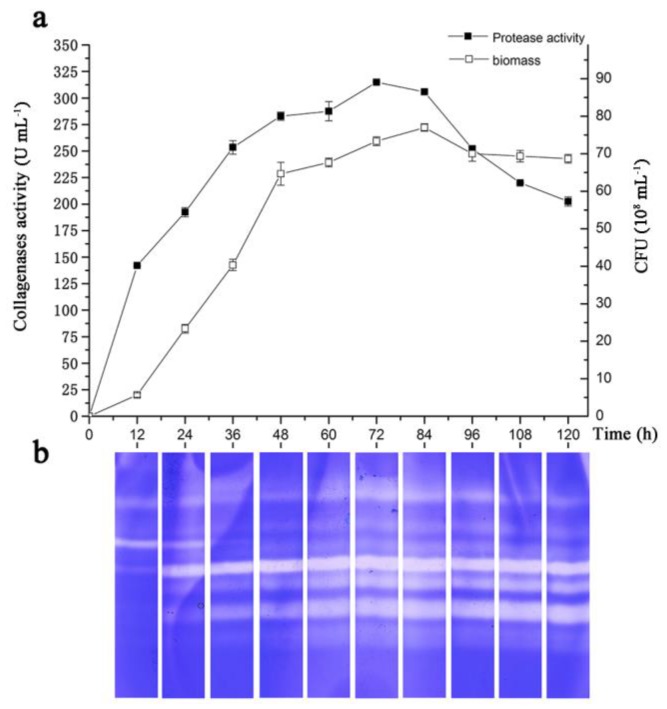
Time course of collagenase yield vs. biomass of *Ps* sp. SJN2 (**a**) and gelatine-immersing zymography (**b**).

The validation of the statistical model and the regression equation was conducted using the optimized conditions. The predicted response for collagenase production was 306.68 U·mL^−1^, and the observed experimental value was 322.58 U·mL^−1^. The experimental production was close to the predicted response, and the yield of collagenases from *Ps* sp. SJN2 increased 2.2-fold compared to the yield from the original fermentation scheme. The optimal fermentation scheme was further examined by the continuous determination of collagenase activity by bacterial biomass evaluation and gelatine-immersing zymography, as shown in Figure 4.

As shown in Figure 4a, the activity of the collagenases from *Ps* sp. SJN2 increased steadily as *Ps* sp. SJN2 grew in the logarithmic-growth phase, and the activity was highest when the biomass entered the stationary phase at approximately 72 h of cultivation time. Then, within approximately 24 h, the activity of the collagenases from *Ps* sp. SJN2 started to decrease, which may be explained by the decrease in biomass over the same period or by transformation of part of collagenases into other non-catalytic proteins. This finding affirmed that collagenase activity is related to biomass, which is characterized by the phenomenon that collagenase activity and biomass both exhibit maximum values at approximately 3.5 d, which is in agreement with the optimal model prediction.

In Figure 4b, the gelatine-substrate immersing zymography validation showed that when the proteases reach a maximum yield at 3.5 d, which was also proven by the time course experiment, there were eight bright strips, which represented activated zymogen and proteases (primarily metalloproteases and serine proteases; Figure S2, Supplementary Material) that might possess the ability to hydrolyze collagen, which proved that the increased activity of the crude enzyme under optimized conditions was caused by the increased yield. Furthermore, the visible strips of proteases with high molecular weight grew progressively darker over culture time, which might partly be due to enzyme autolysis into other mature forms.

### 2.6. Antioxidant Activity of the Hydrolysates

Five kinds of collagen were successfully extracted from seafood waste (salmon skin, seabream scale and Spanish mackerel fish bone), octopus flesh and porcine skin, as shown in Figure 2e. After hydrolysis, the collagen peptides were tested for potential antioxidant activity by DPPH scavenging (Figure 2d) and ORAC assays (Figure 2f–i).

The scavenging of nitrogen radicals released by DPPH was affected by the concentration of collagen peptides; the group of 0.4 mg·mL^−1^ reaction concentrations of collagen (black) has a higher rate than the group of 0.2 mg·mL^−1^ (blue). Furthermore, the collagen peptides from Spanish mackerel bone showed a strong scavenging rate, more than 50%, compared with the positive control, 0.1 mg·mL^−1^ vitamin C, at 73%, while the porcine collagen peptides are lower than 10%.

The ORAC assay was performed using four kinds of marine collagen peptides hydrolysed by collagenases from *Ps* sp. SJN2. The results are shown in Figure 2f–i: the red curve is the positive control, 100 μg·mL^−1^ vitamin C, and the black curve is the negative control, PBS (0.01 M). It was noticed that all the marine peptides have the capacity to absorb the oxygen radical released by AAPH (2,2′-Azobis(2-methylpropionamidine) dihydrochloride). When the collagen peptides were both at low concentrations (navy curve), the absorption capacities were also weak. The absorption capacity increased as the concentration of the collagen peptides increased, and a strong oxygen radical absorption capacity appeared at 400 μg·mL^−1^ (blue curve) for collagen from seabream scales (g) and octopus flesh (h). In addition, antioxidant activity of marine collagen-derived peptides have been widely reported in recent years, such as those from bluefin leatherjacket, tuna, yellowfin sole, Alaska pollock, halibut, round scad and Pacific hake [18], marine by-products of which have been named as sources of antioxidant peptides. Although the bioactive peptides are encrypted within the protein structure, enzymatic hydrolysis could be a useful method to obtain the peptides naturally in an environmentally-friendly, safe and efficient manner. 

## 3. Materials and Methods

### 3.1. Microorganism

The strain *Pseudoalteromonas* sp. SJN2 used in this study was originally isolated from the inshore environment of the South China Sea (18°29′198″ N, 109°34′761″ E). *Ps* sp. SJN2 can produce certain extracellular collagenases. SJN2 was maintained on 2216E agar slants and stored at 4 °C. See the Supplementary Material for descriptions of the raw materials and inoculum preparation.

### 3.2. Fermentation

Bench-scale fermentation was performed in 250mL Erlenmeyer flasks containing different concentrations of raw solution (Supplementary Material) and media components, which were tested according to the statistical experimental design. Flasks were inoculated with seed culture and incubated at 16 °C for 96 h on a rotary shaker at 180 rpm. After fermentation, the crude enzyme was purified by centrifugation at 10,000× *g* for 10 min. Fermentation was carried out in triplicate, and the results represent the average of three trials.

### 3.3. Assay of Collagenase Activity and Substrate Immersing Zymography

The collagenolytic activities against bovine collagen were determined using the method provided by Worthington Biochemical Co. (Lakewood, NJ, USA). The reaction time was 5 h for bovine insoluble type I collagen fiber. For insoluble collagenases, one unit of enzyme releases 1 μmol of Leucine equivalents from collagen in 1 h. Substrate immersing zymography was performed as described in our previous studies. The bands from the SDS-PAGE gel were cut separately from each lane, immersed in each of the pre-warmed substrate solutions and incubated for reaction at 37 °C for 1 h. After washing, the gels were stained with 0.1% (*w*/*v*) Coomassie Brilliant Blue R-250 (Sangon, Shanghai, China) for 3 h and then destained with a solution containing 30% ethanol and 70% acetic acid, and destaining was complete when bands indicating proteolytic activity were clearly visible [19].

### 3.4. Screening of Significant Variables by Using Plackett–Burman Designs

The Plackett–Burman experimental design was used to screen the variables that significantly influenced collagenase production; the detailed scheme was designed by using Design-Expert Version 8.0.6 software (Stat-Ease Inc., Minneapolis, MN, USA). The Plackett–Burman design allows the evaluation of many variables, with 1–4 dummy variables reducing the error. Each variable was tested for two contrary levels: +1 as the high level and −1 as the low level; the level parameters should be appropriate selected such that the strain can survive under the conditions prescribed by each level. The Plackett–Burman experimental design is based on the first-order model as Equation (3) [13]:

*Y* = *β_0_* + ∑*β_i_x_i_* (3)

where *Y* is the predicted response (collagenase activity), *β_0_* is the model intercept, *β_i_* is the linear coefficient and *x_i_* is the level of the independent variable. This model identifies the main parameters required for maximal collagenase production. A total of seven variables, namely temperature, initial pH of the medium, seed inoculation, incubation time, corn meal concentration, bran liquid concentration and soybean meal concentration, were chosen for the present study. The factors under investigation and the levels for each factor selected in the Plackett–Burman design are illustrated in Table 1, and the experimental matrix and results are presented in Table 2. The collagenase activity assay was carried out in duplicate, and the average value was calculated as the response *Y*.

### 3.5. Investigation of the Shifts in the Trends of the Significant Variables Using the Path of Steepest Ascent 

Frequently, the initial estimate of the optimal conditions for the system is far from the actual optimal conditions. Before the final response surface analysis, the path of steepest ascent usually appears as a line through the center of the region of interest and is normal to the fitted surface contours [20]. Three significant independent variables were selected based on the results from the Plackett–Burman designs. The direction of the shift in the trend of each variable was determined by the positive or negative of the coefficient estimate listed in Table 3. The step length (△) of the path of steepest ascent was calculated by following Equation (4):

△ = (Average − Base) × Slope × Ratio(4)

In this formula, Average is the average value of the −1 or +1 level of each variable and Slope is equal to the coefficient estimate ratio. The Ratio is an empirical value derived from pre-experimental data in order to obtain a rational numerical variable for operation. 

Based on the physical and chemical characteristics of the bacteria, the steepest ascent model contained four steps. Specific experimental designs for the path of steepest ascent are shown in Table 4.

### 3.6. Optimization by Response Surface Methodology

Based on the significant variables chosen by the Plackett–Burman design experiment and the operating conditions selected by the steepest ascent experiment, response surface methodology was applied for the augmentation of collagenase production using a central composite design (CCD) [21]. There are five levels (−1.68, −1, 0, +1 and +1.68) estimated for each variable in the CCD design based on the coded values and actual values, as listed in Table 5. The significant variables and their 0-level values were as follows: temperature (16 °C), culture time (3.36 d) and soybean concentration (33.0 g·L^−1^). The +1.68-level value was calculated as the value of the 0-level plus the product of the step unit multiplied by +1.68. A total of 20 combined experiments was performed in triplicate and repeated twice. The complete experimental plan, including the RSM design and collagenase production as the corresponding response value (*Y*), are shown in Table 6. The statistical software package Design-Expert Version 8.0.6 was used to analyze the design.

### 3.7. Statistical Analysis

The data obtained from the central composite designs with three factors (temperature, culture time and soybean powder concentration) were used to perform analysis of variance (ANOVA) with Design-Expert Version 8.0.6 software, as presented in Table 7. After completing the response surface methodology experiments and measuring the collagenase yield, the response surface regression procedure was used to fit the experimental results from the RSM to the following second-order polynomial regression Equation (5) [21,22]:

(5) $$Y=\beta_{0}+\underset{i}{\sum }\beta_{i}X_{i}+\underset{\mathrm{ii}}{\sum }\beta_{\mathrm{ii}}X_{i}^{2}+\underset{\mathrm{ij}}{\sum }\beta_{\mathrm{ij}}X_{i}X_{j}$$

where *Y* is the predicted response value (collagenase production), β_0_ is the center point of the system, β*_i_* is the linear coefficient, β*_ii_* is the quadratic coefficient and β*_ij_* is the interaction coefficient, while *X_i_*, *X_i_*^2^ and *X_j_* are the linear, quadratic and interaction terms of the independent variables, respectively. The fitted model was then expressed as three-dimensional surface and contour plots to describe the relationship between the responses and the experimental levels of each of the variables studied. 

### 3.8. Validation of the Optimization Model

The optimization model was validated by adjusting three parameters for each of the variables. A triplicate culture set was grown under experimental conditions derived from the optimization scheme, and collagenase production was compared to the predicted response value.

For further validation, a time-course experiment for collagenase production and biomass fluctuation was conducted, and gelatine-immersing zymography was also performed. Continuous fermentation was performed under the optimal conditions, and collagenase production and biomass were recorded [23,24]. Changes in extracellular collagenase secretion were detected by non-denaturing gel electrophoresis with gelatine as the immersing substrate of soluble collagen analogues [19]. The collagenase activity was measured, and the biomass was quantified using the spread plate method.

### 3.9. Purification of Ps sp. SJN2 Collagenases

The total extracellular collagenases from the fermentation broth were extracted by ammonium sulfate precipitation. The fermentation liquid was centrifuged at 10,000 rpm for 30 min at 4 °C. Ammonium sulfate was added slowly up to 40% (*w*/*w*), and the solution was allowed to stand overnight at 4 °C. The precipitate was collected and redissolved in water. The supernatant, containing active collagenases, was dialyzed in Tris-HCl buffer (pH 8.8, 20 mM) at 4 °C overnight.

The fraction containing active collagenases (Col SJN2) in the dialysis fluid was further purified on a HiTrap Capto DEAE (GE Healthcare, Boston, MA, USA) column with an NGC chromatography system (Bio-Rad, Hercules, CA, USA) after filtration through a 0.45-μm filter membrane. The column was equilibrated with Tris-HCl buffer (pH 8.8, 20 mM). Then, 5 mL of Col SJN2 were loaded onto the pre-equilibrated column at a flow rate of 1.0 mL·min^−1^ and washed with buffer Tris-HCl for 10 min. Then, elution was conducted using a linear gradient of 1 M NaCl (0–100%) at a flow rate of 2.0 mL·min^−1^. Fractions were monitored at 280 nm, and the Col SJN2 fraction containing active collagenases was centrifuged in an ultrafiltration tube with a 3-kDa molecular weight cut-off (Millipore, Temecula, CA, USA) at 5000× *g* for 30 min at 4 °C. The portion of the Col SJN2 fraction with molecular weight higher than 3 kDa was further purified by size exclusion chromatography.

Col SJN2 was then loaded onto a Superdex-200 (GE Healthcare, Boston, MA, USA) column pre-equilibrated with distilled water. Elution was performed using distilled water at a flow rate of 0.5 mL·min^−1^. Fractions were monitored at 280 nm, and the fractions with collagenase activity were stored.

### 3.10. Preparation of Collagen from Fishery By-Products

To study the enzymatic properties of collagenases [25] from *Ps* sp. SJN2, five kinds of native collagen were extracted from porcine skin, octopus flesh and fishery by-products (salmon fish skins, seabream fish scales and Spanish mackerel fish bones). Extraction methods are described in the Supplementary Material.

### 3.11. Enzymatic Hydrolysis and Properties of Ps sp. SJN2 Collagenase

Extracted collagen was digested with a crude protease of *Ps* sp. SJN2 at an enzyme-to-substrate ratio of 1:10 (*v* (mL)/*w* (mg)). The reaction was carried out at 45 °C in PBS (pH 7.0, 0.1 M) for 1 hour and stopped by incubation at 95 °C for 10 min. The resultant slurry was collected and centrifuged at 10,000× *g* at 4 °C for 10 min. The supernatant was collected and stored at 4 °C until further analysis.

Substrate-immersing zymography was conducted to compare the properties of the crude enzymes from *Ps* sp. SJN2, *Ps* sp. SBN2-2, *Ps* sp. SGS2-2, *Ps* sp. SWN-1, *Ps* sp. SWN-2, *Vibrio* sp. HK3-2, *Vibrio* sp. SJN4 and *Planococcus* sp. SYT1.

Additionally, the effects of different concentrations of ions on the enzymatic activity of *Ps* sp. SJN2 collagenase were tested. Different ions (Cd^2+^, Mn^2+^, Ca^2+^, Fe^3+^, Ag^+^, Al^3+^, Ba^2+^, Cu^2+^, Zn^2+^, Mg^2+^, Fe^2+^ and Co^2+^) and the metal-chelating agent EDTA were added to the reaction buffer containing crude enzyme at concentrations of 2 mM and 10 mM, and the specific activity of the collagenase was measured.

The collagen-swelling effect was also observed under SEM. A total of 5 mg of type I insoluble collagen was mixed with 2 mL of 20 mM PBS (pH 8.5) containing 200 μL of *Ps* sp. SJN2 collagenase. The samples were incubated at 37 °C for 12 h with continuous stirring. The samples were observed using scanning electron microscopy (FEI, Hillsboro, OR, USA).

### 3.12. DPPH Radical Scavenging and ORAC Assay

A modified DPPH radical scavenging assay was performed [26,27]. Ethanolic solutions of DPPH (10^−4^) and collagen hydrolysates were mixed in disposable Eppendorf tubes so that the final mass ratios of hydrolysates to DPPH were 1:5. The samples were sealed and incubated for 60 min in the dark at 37 °C, and the decrease in absorbance at 517 nm was measured against ethanol in 96-well plates using an Enspire spectrophotometer (Perkin Elmer, Waltham, MA, USA). Vitamin C was used as a positive control. All determinations were performed in triplicate. 

The ORAC assay was performed based on the method described by Zulueta [28], with some modifications. In this system, AAPH is the source of free radicals that can attack the fluorescein and lead to fluorescence decay. The reaction was carried out in 75 mM phosphate buffer (pH 7.4). Sample solution (20 μL) and fluorescein (150 μL, 96 nM) were added into a 96-well plate and pre-incubated at 37 °C in the Enspire spectrophotometer. The reaction was initiated by adding 30 μL of AAPH (320 mM). The reaction was performed at 37 °C. The fluorescence intensity [29] was measured every 60 s for 120 cycles with excitation and emission wavelengths of 485 nm and 538 nm, respectively. The positive control was vitamin C, which was used at the same concentration as in the DPPH radical scavenging assay.

## 4. Conclusions

Compared with other native bacteria, the extracellular collagenases of *Ps* sp. SJN2, composed of proteases (e.g., metalloproteases and serine proteases) with different molecular weights, showed higher enzyme activity towards porcine, bovine and marine collagen. Given the increasing economic relevance of marine collagenases, this study was conducted to optimize a variety of fermentation parameters, including medium composition and culture conditions, for maximal collagenase production. The results indicated that the Plackett–Burman design and response surface methodology are effective and efficient approaches to improve collagenase production via optimization of *Ps* sp. SJN2 fermentation conditions. The maximum collagenase yield of 322.58 ± 9.61 U·mL^−1^ was achieved under the optimized conditions, which was in agreement with the production value of 306.68 U·mL^−1^ predicted by the model. Finally, for optimal fermentation, the cells were cultured at 17.32 °C for 3.72 d in a medium containing soybean 34.23 g·L^−1^, corn meal 30 g·L^−1^ and bran liquid 15 mL·L^−1^ at an initial pH value of 8.5 and an inoculum volume of 1% (*v*/*v*). Significant improvement (2.2-fold) in the production of collagenases by *Ps* sp. SJN2 was accomplished. In addition, the marine collagen hydrolysed by collagenases from *Ps* sp. SJN2, especially those from Spanish mackerel fish bone and seabream fish scale, showed better prospects for application as antioxidant peptides. Above all, the optimized medium established in this study could provide a basis for further study of large-scale fermentation of *Ps* sp. SJN2 for marine collagenase yield improvement.

## Figures and Tables

**Table 1:** Variables tested for collagenase production using Plackett–Burman designs and the levels for each variable.

Variables	Component	Unit	Lower Level (−1)	Higher Level (+1)
*X_1_*	Temperature	°C	18.0	27.0
*X_2_*	pH	-	7.5	8.5
*X_3_*	Inoculum	μL	500.0	750.0
*X_4_*	Culture time	d	4.0	6.0
*X_5_*	Corn meal	g·L^−1^	20.0	30.0
*X_6_*	Bran liquid	mL·L^−1^	10.0	15.0
*X_7_*	Soybean powder	g·L^−1^	20.0	30.0
*X_8_*	Dummy variable	-	-	-
*X_9_*	Dummy variable	-	-	-
*X_10_*	Dummy variable	-	-	-
*X_11_*	Dummy variable	-	-	-

**Table 2:** Plackett–Burman design matrix with corresponding results.

Run	Variables											Collagenase Activity U·mL^−1^
	*X_1_*	*X_2_*	*X_3_*	*X_4_*	*X_5_*	*X_6_*	*X_7_*	*X_8_*	*X_9_*	*X_10_*	*X_11_*	
1	−1	+1	−1	+1	+1	−1	+1	+1	+1	−1	−1	230.53
2	−1	−1	−1	+1	−1	+1	+1	−1	+1	+1	+1	235.50
3	−1	+1	+1	−1	+1	+1	+1	−1	−1	−1	+1	273.33
4	+1	−1	+1	+1	−1	+1	+1	+1	−1	−1	−1	162.23
5	+1	+1	+1	−1	−1	−1	+1	−1	+1	+1	−1	168.39
6	+1	−1	+1	+1	+1	−1	−1	−1	+1	−1	+1	127.82
7	+1	−1	−1	−1	+1	−1	+1	+1	−1	+1	+1	225.40
8	+1	+1	−1	−1	−1	+1	−1	+1	+1	−1	+1	202.29
9	−1	−1	−1	−1	−1	−1	−1	−1	−1	−1	−1	215.47
10	−1	−1	+1	−1	+1	+1	−1	+1	+1	+1	−1	218.72
11	+1	+1	−1	+1	+1	+1	−1	−1	−1	+1	−1	147.17
12	−1	+1	+1	+1	−1	−1	−1	+1	−1	+1	+1	173.70

**Table 3:** Variance analysis of the Plackett–Burman linear model.

Source	df	Collagenase Activity of Fermented Broth ^a^				
		Sum of Squares	Coefficient Estimate	Mean Square	*F*-Value	*p*-value Probability > F
Model ^a^	7	18,796.53	-	2685.22	9.770	0.0219 ^b^
*X_1_*	1	8213.77	−26.16	8213.77	29.880	0.0054 ^b^
*X_2_*	1	8.79	0.86	8.79	0.032	0.8668
*X_3_*	1	1455.41	−11.01	1455.41	5.290	0.0829
*X_4_*	1	4280.74	−18.89	4280.74	15.570	0.0169 ^b^
*X_5_*	1	356.35	5.45	356.35	1.300	0.3184
*X_6_*	1	799.01	8.16	799.01	2.910	0.1634
*X_7_*	1	3682.46	17.52	3682.46	13.400	0.0216 ^b^
Residual	4	1099.59	-	274.90	-	-
Corrected Total	11	19,896.12	-	-	-	-

^a^ *R*^2^ = 0.9447. ^b^ Model terms are significant.

**Table 4:** Experimental details and results of the steepest ascent design with four steps approaching the response region.

Item	Temperature (°C)	Culture time (d)	Soybean Concentration (g·L^−1^)	Collagenase Activity (U·mL^−1^)
Slope ^a^	−26.16	−18.89	17.52	-
Base ^b^	18.00	4.00	30.00	-
Average ^c^	22.50	5.00	25.00	-
Ratio ^d^	0.017	0.034	0.034	-
△ ^e^	−2.00	−0.64	3.00	-
Base	18.00	4.00	30.00	224.66 ± 11.53
Base+△	16.00	3.36	33.00	279.18 ± 8.71
Base+2△	14.00	2.72	36.00	206.22 ± 26.47
Base+3△	12.00	2.08	39.00	195.09 ± 9.65

^a^ Coefficient estimate in Table 3. ^b^ −1/+1 level in the Plackett–Burman design in Table 1. ^c^ Average value of the −1 level and +1 level in Table 1. ^d^ An appropriate ratio determined by the experimenter, based on experiential knowledge and laboratory conditions, to reasonably adjust the step coefficient in this model. ^e^ Step length, calculated as Equation (4).

**Table 5:** Levels of significant variables for the response surface design.

Variables	Component	Unit	+1.68 Level	+1 Level	0 Level	−1 Level	−1.68 Level
*A*	Temperature	°C	19.36	18.00	16.00	14.00	12.64
*B*	Culture time	d	4.44	4.00	3.36	2.72	2.28
*C*	Soybean powder	g·L^−1^	38.04	36.00	33.00	30.00	27.96

**Table 6:** The matrix of the response surface experiment and the corresponding results.

Run	Variables			Collagenase Activity (U·mL^−1^)
	*A*	*B*	*C*	
1	+1.68	0	0	251.13
2	+1	−1	+1	221.71
3	0	−1.68	0	187.44
4	+1	−1	−1	160.45
5	−1	+1	−1	123.73
6	0	0	0	278.35
7	0	0	0	282.97
8	+1	+1	−1	197.21
9	+1	+1	+1	302.56
10	0	+1.68	0	243.74
11	−1.68	0	0	182.53
12	0	0	0	278.07
13	0	0	0	287.85
14	0	0	−1.68	107.18
15	−1	+1	+1	214.38
16	0	0	+1.68	192.30
17	−1	−1	+1	182.51
18	−1	−1	−1	119.22
19	0	0	0	280.56
20	0	0	0	319.77

**Table 7:** Variance analysis of the response surface methodology.

Source	df	Collagenase Activity of Fermented Brotha ^a^			
		Sum of Squares	Mean Square	*F*-Value	*p*-value Probability > *F*
Model	9	75,803.27	8422.59	35.19	0.0001 ^b^
*A*	1	9356.35	9356.35	39.09	0.0001 ^b^
*B*	1	4528.07	4528.07	18.92	0.0014 ^b^
*C*	1	15,744.59	15,744.59	65.78	0.0001 ^b^
*AB*	1	824.79	824.79	3.45	0.0931
*AC*	1	20.07	20.07	0.084	0.7781
*BC*	1	638.14	638.14	2.67	0.1335
*A*^2^	1	8823.57	8823.57	36.87	0.0001 ^b^
*B*^2^	1	9139.01	9139.01	38.18	0.0001 ^b^
*C*^2^	1	33,848.58	33,848.58	141.43	0.0001 ^b^
Residual	10	2393.38	239.34	-	-
Lack of Fit	5	1111.68	222.34	0.87	0.5601
Pure Error	5	1281.71	256.34	-	-
Corrected Total	19	78,196.66	-	-	-

^a^ *R*^2^ = 0.9694, C.V. = 7.01%. ^b^ Model terms are significant.

## Supplementary Materials

The following are available online at www.mdpi.com/1660-3397/15/12/377/s1: Figure S1: Gelatin immersing zymography of Col SJN2, Figure S2: Crude enzyme zymography of *Ps* sp. SJN2, Table S1: Collagenases activity in purification process. Supporting information about the methods of inoculum preparation for fermentation and preparation of collagen from fishery by-products is provided in the Supplementary Material.
