# Supplementary material for: Optimization of Collagenase Production by Pseudoalteromonas sp. SJN2 and Application of Collagenases in the Preparation of Antioxidative Hydrolysates
Source: Mar Drugs. 2017 Dec 2;15(12):377. doi: 10.3390/md15120377 (PMC5742837; doi:10.3390/md15120377)
Supplement: Supplementary file 1 [file marinedrugs-15-00377-s001.pdf]

Supplementary Material for

Optimization of collagenase production by *Pseudoalteromonas* sp. SJN2 and application of collagenases in the preparation of antioxidative hydrolysates

XingHao Yang[1,2,†], Xiao Xiao[1,†], Dan Liu[1], RiBang Wu[1], CuiLing Wu[1], Jiang Zhang[1], JiaFeng Huang[1], BinQiang Liao[1] and HaiLun He[1,*]

[1] School of Life Sciences, Central South University, Changsha 410013, China
[2] Hunan Bailin Biological Technology Incorporated Company, Changsha 410205, China
* Correspondence: helenhe@csu.edu.cn; Tel.: +86-0731-82650230
[†]These authors contributed equally to this work.

* Correspondence: helenhe@csu.edu.cn; Tel.: +86-0731-82650230

Results

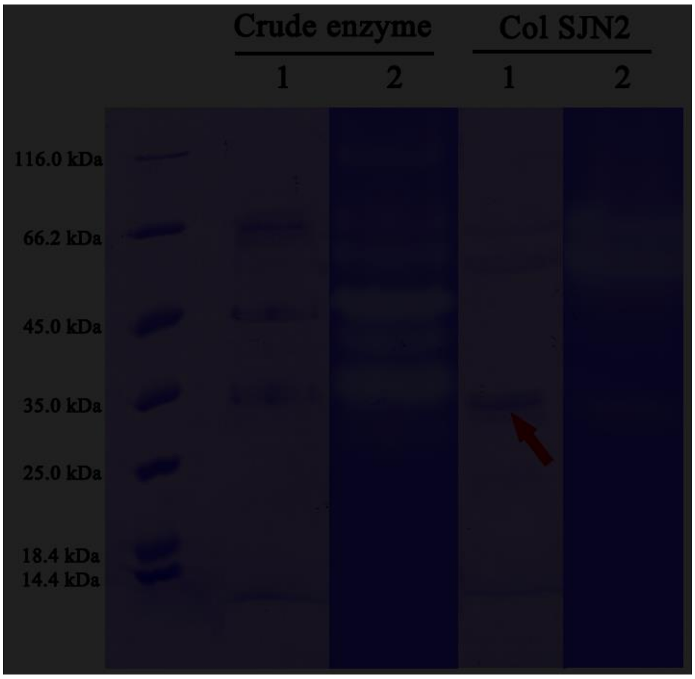

Figure S1. Gelatin immersing zymography of Col SJN2. Line marked 1: non-denaturalization SDS-polyacrylamide gel (non-boiled samples, remained catalytic activity); line marked 2: gelatin immersing zymography.

Table S1. Collagenases activity in purification process

No.	Purification stage	Total collagenases activity (U)	Total protein (mg)	Specific activity (U mg^{-1})
1	Crude enzyme	320,000	60	5,333.3
2	Ammonium sulfate precipitation	99,200	13.8	7,188.4
3	Anion exchange	16,320	0.48	34,000.0
4	Size exclusion	11,750	0.19	61,842.1

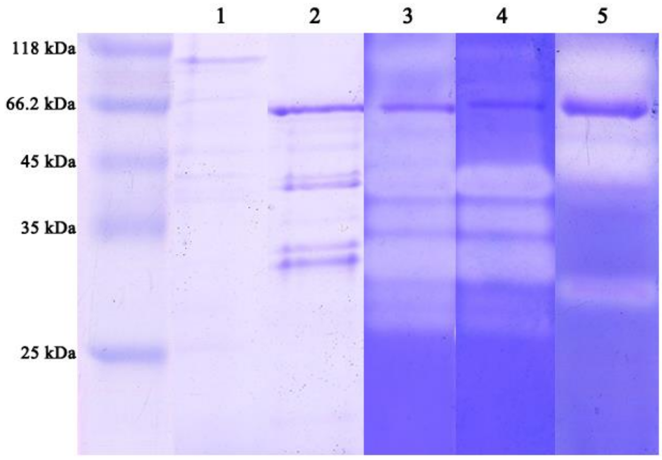

Figure S2. Crude enzyme zymography of *Ps* sp. SJN2. Line 1: SDS-polyacrylamide gel; line 2: non-denaturalization SDS-polyacrylamide gel (non-boiled samples, remained catalytic activity); line 3: gelatin immersing zymography; line 4: gelatin immersing zymography with OP (1, 10-Phenanthroline monohydrate, 10 mM); line 5: gelatin immersing zymography with PMSF (Phenylmethanesulfonyl fluoride, 10mM). The immersing zymography of crude enzyme with gelatin, OP and PMSF showed that gelatinases in crude enzyme (line 3), might possess collagen-hydrolysis ability, could be partially inhibited by OP (line 4) and PMSF (line 5). Metalloproteases and serine proteases are the major enzymes in crude enzyme of *Ps* sp. SJN2.

Raw Materials

All microbiological media components were purchased from Klontech (JiNan, China). Bran, corn meal and soybean powder were purchased from supermarket. In order to guarantee the quantitative nutritive composition, bran was boiled in a volume of double distilled water for about 30 minutes. Then the solution was filtered as bran liquid prepared for fermentation. The collagenases from *Clostridum histolyticum* (Col H) was purchased from Sangon Biotech (Shanghai) Co., Ltd. 1,1-diphenyl-2-picrylhydrazyl (DPPH), fluorescein, 2,2'-Azobis(2-methylpropionamidine)dihydrochloride (AAPH) and Vitamin C were purchased from Sigma-Aldrich China Ltd.

Raw solution contained a variety of ingredients about 0.1 % (w/w) Na_2HPO_4, 0.03 % (w/w) KH_2PO_4, 0.1 % (w/w) $CaCl_2$ and 0.1 % (w/w) Na_2CO_3 dissolved in artificial seawater (28.15 g NaCl, 6.92 g $MgSO_4 \cdot 7H_2O$, 0.67 g KCl , 5.51 g $MgCl \cdot 6H_2O$ and 1.45 g $CaCl_2 \cdot H_2O$ per liter of distilled water). Raw solution was used to dissolve fermentation medium components which were further optimized in Methods.

Inoculum preparation

The strain cells obtained from the 2216E agar slants were inoculated into 50 ml of liquid 2216E medium in an Erlenmeyer flask, and incubated at 16 °C for about 16 h with shaking at 180 rpm. The culture broth, with bacterium fluid OD_{600} = 0.8±0.2 was served as seed culture for all following experimental designs.

Methods

Preparation of collagen from fishery by-products.

The octopus flesh was cut into small cubes, followed 10 times volume of propanol soaking for 3 days, then filtered through sterile gauze and the flesh cubes were collected. Wash several times with distilled water before soaked in 10 times volume of NaOH (0.5 M) for 3 days. Filtered and washed pH to neutral, the flesh cubes were then immersed with 2 times volume of glacial acetic acid (0.5M) for 3 days. The solution was centrifuged at 1000 rpm for 10 min and the supernatant was collected. Added NaCl to final concentration 10% (m/v), rested in 4°C for 24 h. The collagen was separated out, collected the sediment after centrifuged at 10000 rpm, 4°C for 15 min. Using PBS (0.01 M, pH 7.4) dissolving the precipitation and the extraction was detected by SDS-PAGE electrophoresis.

The porcine skin collagen and salmon fish skin collagen were extracted as the same protocol mentioned above.

The seabream fish scales were first washed and cut into small pieces. Then added 5 times volume of distilled water and boiled at 70°C for 5 min with continuous whisking. Filtered and collected the solution and then centrifuged at 8000 rpm for 20 min. The supernatant contained the fish scale collagen, optional vacuum freeze-drying or directly stored at -20°C.

The spanish mackerel fish bone was washed and completely chopped into short pieces after removed of flesh attached. Then the bone pieces were immersed with 20 times volume NaOH (0.1 M) for 4 h. Filtered through a sieve and washed the bone

pieces pH to neutral. Soaked in 5 times volume of EDTA (0.5 M) for 5 d, 4°C, EDTA solution was daily changed. Filtered and added 20 times volume of 10% isopropanol, rested in 4°C for 1 d. Then the bone pieces were filtered and washed pH to neutral. Equivalent glacial acetic acid was added and soaked for 3 d, 4°C. The solution was collected and added NaCl to final concentration 0.9 M. The collagen was then appeared as white flocculent precipitate. The deposition was collected by centrifuge at 10000 rpm for 15min, and dissolved with PBS. All the collagens had been quantitatively tested by Bradford and prepared for enzymatic hydrolysis.
